# Controlled Surface Engineering of Chitosan Hydrogels: Alkali/Urea Dissolution for Ratio-Specific Neodymium and Praseodymium Recovery

**DOI:** 10.3390/polym17192567

**Published:** 2025-09-23

**Authors:** John Earwood, Baolin Deng

**Affiliations:** 1 Department of Civil & Environmental Engineering, University of Missouri, Columbia, MO 65211, USA; jen9y@umsystem.edu; 2 Missouri Water Center, Columbia, MO 65211, USA

**Keywords:** rare earth element recovery, NdPr extraction, ion-imprinted polymers, chitosan hydrogels, selective uptake

## Abstract

Rare earth elements (REEs) are critical for advanced technologies, with neodymium and praseodymium being essential to high-performance permanent magnets. The separation of these adjacent lanthanides represents a significant challenge due to their nearly identical chemical properties, with traditional chitosan surfaces exhibiting limited discrimination between chemically similar elements. Current separation methods require multiple processing steps and cannot maintain predetermined compositional ratios. Engineered polymer interfaces with controlled binding site distribution represents a critical advancement for selective separation, but achieving ratio-controlled extraction of adjacent elements remains challenging. Here, we demonstrate a novel interface engineering approach using alkali/urea dissolution to restructure chitosan networks, creating dual-template alkali/urea chitosan hydrogels (NdPr-AUCH) for simultaneous selective co-extraction of Nd(III) and Pr(III). We show that the dissolution–reformation process enables templated Nd:Pr selectivity ratios (1:1, 2:1, and 4:1) that directly correspond to synthesis compositions. NdPr-AUCH-11 achieved maximum uptake capacities of 19.85 mg/g for Nd(III) and 16.89 mg/g for Pr(III), while NdPr-AUCH-41 maintained 3.07:1 Nd:Pr selectivity in competitive environments. Thermodynamic analyses reveal consistently lower energy requirements for Nd(III) binding compared to Pr(III), demonstrating how interface engineering amplifies coordination differences between adjacent lanthanides. This work represents the first demonstration of ratio-controlled extraction of adjacent lanthanides within a single polymer matrix, advancing interface-engineered materials for selective rare earth recovery.

## 1. Introduction

Rare earth elements (REEs) play a crucial role in many modern technologies, from renewable energy to advanced electronics and defense applications [1,2,3,4]. The precise ratio of REEs determines material performance across applications, with specific combinations essential for optimized magnets, catalysts, phosphors, and electronics [5,6]. Among these strategic compositions, neodymium (Nd) and praseodymium (Pr) are vital to the production of high-performance permanent magnets. The incorporation of Pr in NdFeB magnets can reduce production costs while maintaining functionality [7]. Modern commercial NdFeB magnets typically contain Nd:Pr ratios ranging from 3:1 to 4:1. This “didymium” mixture (Nd-Pr) has become increasingly important in the permanent magnet industry, particularly as manufacturers seek to optimize cost–performance ratios [8].

The selective separation of chemically similar lanthanides, particularly adjacent elements, represents one of the most challenging aspects of REE processing [9]. These elements’ nearly identical ionic radii (0.995 Å and 1.013 Å) and chemical properties make their separation a significant bottleneck in the REE supply chain, typically requiring multiple stages of solvent extraction with precise pH control [10,11]. Direct extraction of didymium (a mixture of praseodymium and neodymium) and other vital lanthanides at strategic REE ratios would eliminate several processing steps, reducing costs while enabling precise compositional control for magnet applications.

Recent advancements in selective extraction methods have shown promise in addressing challenges in one-step dual REE separation [12,13,14]. Interpolymer systems composed of industrial sorbents have demonstrated separation of neodymium from praseodymium in mixed solutions [15]. Novel pyridine Schiff base compounds extracted both neodymium and praseodymium from NdFeB magnet waste with high sorption capacities [7]. In the field of imprinted materials, dual-ion-imprinted thermosensitive chitosan hydrogels have demonstrated effective separation of lanthanum and yttrium [16], while dual-template docking oriented ionic imprinted films have shown potential for the simultaneous recovery of neodymium and dysprosium from acidic solutions [17]. Despite these advances, no approach has achieved ratio-controlled extraction of adjacent lanthanides, which would enable direct access to commercially relevant didymium REE compositions without requiring additional processing steps.

The development of engineered polymer interfaces with uniform binding site distribution represents a critical step toward overcoming these fundamental limitations. Previous dual-template systems have targeted lanthanides with significant size or charge differences, whereas Nd^3+^ and Pr^3+^ differ by only 0.008 Å. This work demonstrates that controlled interface engineering can overcome even these minimal physicochemical differences to achieve programmable selectivity ratios. Specifically, controlled restructuring of polymer surfaces through dissolution and reformation processes creates homogeneous recognition environments where coordination chemistry can dominate over electrostatic effects. In our previous work, we developed a novel interface engineering approach using alkali/urea dissolution to restructure chitosan networks, creating alkali/urea dissolved chitosan-based ion-imprinted hydrogels (AUCH) for the selective extraction of individual REEs from complex matrices [18]. We hypothesize that this dissolution–reformation process transforms the heterogeneous chitosan surface into a uniform network structure with controlled binding site distribution, enabling the amplification of subtle coordination differences between Nd^3+^ and Pr^3+^. Expanding on this platform, we report novel dual-template AUCH materials designed to maintain specific Nd:Pr ratios (1:1, 2:1, and 4:1) that directly correspond to commercially relevant didymium compositions. This approach represents, to our knowledge, the first demonstration of imprinted materials capable of ratio-controlled extraction of adjacent lanthanides within a single polymer matrix, even in the presence of competing ions.

## 2. Materials and Methods

Neodymium (III) chloride hexahydrate and praseodymium (III) chloride hexahydrate (both 99.9% trace metals basis), 1,2,7,8-diepoxyoctane (DEO, 97%), a rare earth element standards mix for inductively coupled plasma (ICP) analysis (16 elements, 50 mg/L in nitric acid), dysprosium (III) chloride hexahydrate, terbium (III) chloride hexahydrate (all 99.9% trace metals basis), and low molecular weight chitosan (deacetylated chitin) were all purchased from Sigma-Aldrich (St. Louis, MO, USA). Lithium hydroxide (anhydrous powder), potassium hydroxide (pellets), urea (certified ACS), and ethylenediaminetetraacetic acid (EDTA, ACS reagent) were obtained from Fisher Scientific (Hampton, NH, USA). Additional lanthanide salts including samarium (III) chloride hexahydrate (99%), lanthanum (III) chloride heptahydrate (ACS reagent), and europium (III) chloride hexahydrate (99.9% trace metals basis) were sourced from Sigma-Aldrich. All other chemicals and reagents were of analytical grade and used without further purification. Ultrapure Milli-Q 18.2 MΩ-cm deionized water was used throughout all experiments.

### 2.1. Synthesis of Dual-Template Alkali/Urea Chitosan Ion-Imprinted Hydrogels (NdPr-AUCH)

An aqueous solution containing LiOH/KOH/Urea/H_2_O was prepared as a solvent for chitosan dissolution under stirring, following our established protocol [18]. Chitosan (5% *w*/*w*) was added to the solvent, producing a slurry that was subjected to freezing at −30 °C overnight. The resulting thawed chitosan slurry was used for subsequent processing steps.

For dual-template synthesis, three distinct imprinting solutions were prepared with effective Nd:Pr molar ratios of 1:1, 2:1, and 4:1. Each imprinting solution was prepared by dissolving appropriate amounts of NdCl_3_·6H_2_O and PrCl_3_·6H_2_O in ultrapure deionized water to achieve a total metal concentration of 6.67% *w*/*w*. The specific imprinting solutions were then added to separate batches of the thawed chitosan slurry and mixed thoroughly, resulting in a final LiOH/KOH/Urea/H_2_O weight ratio of 4.5:7:8:80.5.

The prepared slurries were centrifuged at 10,000 rpm for 5 min at 5 °C to remove air bubbles and separate any undissolved chitosan particles or aggregates, ensuring only the fully dissolved chitosan solution was used for hydrogel preparation. The transparent chitosan solutions were then spread on glass plates and cast to a thickness of 100 μm using a casting knife. Immediately after casting, the glass plates were transferred to a hot water bath maintained at 40 °C and soaked for 30 min.

**Figure 1:**
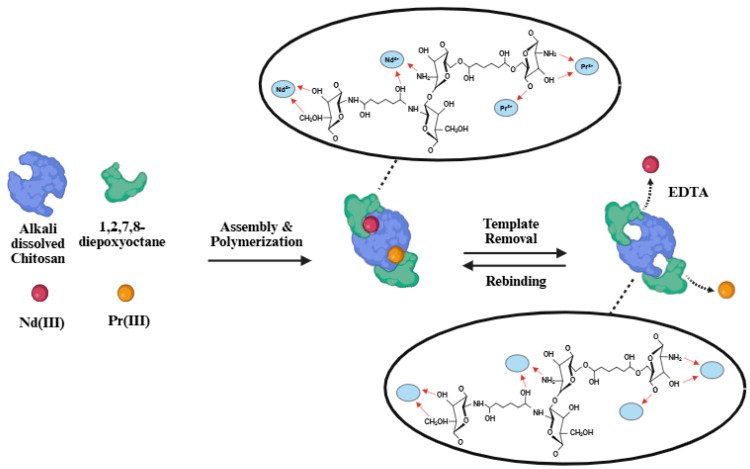
NdPr-AUCH Synthesis Regime.

For 1,2,7,8-diepoxyoctane (DEO) crosslinking, a procedure similar to that reported by Bagheri et al. (2020) was followed [19]. First, deionized water was adjusted to pH 10 and heated to 50 °C before thoroughly dispersing DEO to create a 6 g/L solution. The dual-template AUCH materials were then submerged in the DEO solution for 6 h. After washing with deionized water, the DEO-crosslinked polymers were soaked in 0.05 M EDTA solution overnight (8+ hours) under gentle stirring at room temperature for template removal, producing the NdPr-AUCH materials (NdPr-AUCH-11, NdPr-AUCH-21, and NdPr-AUCH-41 corresponding to 1:1, 2:1, and 4:1 template ratios, respectively) (Figure 1).

### 2.2. Preparation of Control Materials (cAUCH)

Non-imprinted chitosan hydrogels crosslinked with 1,2,7,8-diepoxyoctane (cAUCH) were prepared following the same procedures as their imprinted counterparts, but without the addition of Nd (III) and Pr (III) or subsequent EDTA elution.

### 2.3. Extraction Performance Evaluation

**Figure 2:**
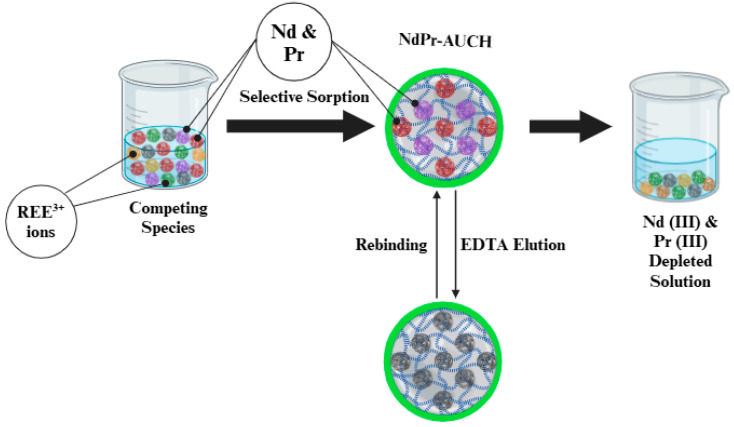
NdPr-AUCH Selective Extraction.

Uptake experiments were conducted in batch to assess the performance of the dual-template AUCH materials. Adsorbents were added to 50 mL of solution in sealed containers, mixing at a constant rate of 80 rpm. The impact of pH on uptake was investigated from pH 3 to 7, with adjustments made using 1.0M HCl and NaOH solutions. Kinetic studies tracked uptake over a 24 h period at optimal pH (pH = 4), with initial Nd:Pr concentrations of 50 mg/L each. Isotherm experiments were performed at optimal pH across a concentration range of 10–75 mg/L Nd:Pr. Both kinetic and isotherm studies were performed at various temperatures, using a temperature-controlled water bath. To evaluate selectivity, multi-component solutions containing 50 mg/L each of Nd(III), Pr(III), La(III), Sm(III), Eu(III), Dy(III), and Tb(III) were used at optimum pH (Figure 2). Regeneration capabilities were assessed through five consecutive uptake-desorption cycles, using 0.05 M EDTA as the eluent. After each uptake period, samples were filtered through 0.45 μm membranes and acidified with 2% HNO_3_ for analysis. Metal ion concentrations were determined using ICP-OES.

### 2.4. Sample Characterization and Analyses

The dual-template AUCH materials were characterized with the following instruments. Surface morphology and elemental composition were examined via SEM (FEI Quanta 600F, FEI Company, Hillsboro, OR, USA) with EDS capabilities. Structural analysis and functional group identification were performed using FTIR-ATR spectroscopy (Nicolet 4700, Thermo Scientific, Waltham, MA, USA). Thermal properties were assessed through DSC-TGA (SDT 600, TA Instruments, New Castle, DE, USA) under nitrogen atmosphere. For quantitative analysis of lanthanide concentrations, an ICP-OES (Thermo Fischer iCAP PRO, Thermo Fischer Scientific, Waltham, MA, USA) was employed, with optimized parameters for adjacent lanthanide detection. Sample preparation involved acidification and filtration, while analysis utilized matrix-matched standards and inter-element corrections to ensure accuracy.

## 3. Results

### 3.1. Surface Morphology Analysis via SEM

**Figure 3:**
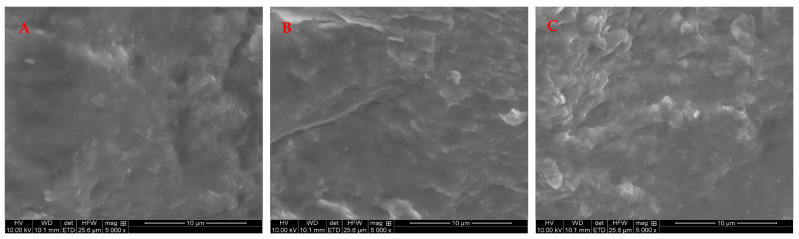
SEM photos of NdPr-AUCH-11 (**A**), NdPr-AUCH-21 (**B**), and NdPr-AUCH-41 (**C**).

The morphology of the dual-templated NdPr-AUCH polymers were investigated using scanning electron microscopy (SEM). As shown in Figure 3, the three polymers exhibit predominantly similar characteristics with smooth surfaces and minor textural features including shallow depressions and protrusions. As Nd:Pr loading increases, a slight progression in surface texture becomes apparent. NdPr-AUCH-11 displays the most uniform surface among the samples, while NdPr-AUCH-21 and NdPr-AUCH-41 show a subtle progressive increase in surface roughness and textural elements. Unlike the significant morphological differences observed between differently crosslinked AUCH materials in previous studies [18], these dual-templated polymers maintain relatively consistent morphology regardless of template ratio. This morphological consistency suggests that varying the Nd:Pr template ratio within the dual-templated system does not substantially alter the overall polymer organization during synthesis, though the minor textural progression may contribute to the selective binding properties observed in adsorption experiments.

### 3.2. Functional Group Analysis via FTIR

**Figure 4:**
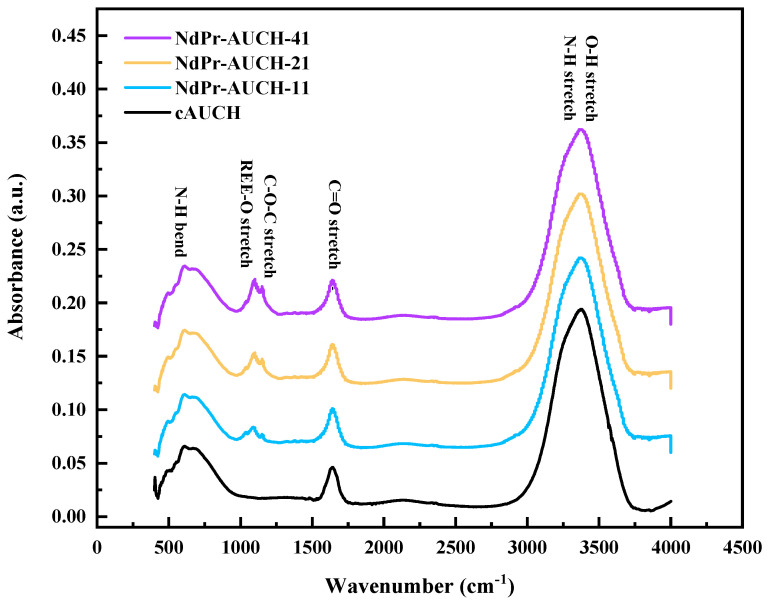
FTIR Spectra of the NdPr-AUCH materials and control polymer.

Figure 4 displays the FTIR spectra of dual-templated Nd:Pr imprinted polymers alongside the control polymer. The broad band observed around 3400–3500 cm^−1^ is attributed to overlapping O-H and N-H stretching vibrations, characteristic of the chitosan backbone. Distinctive peaks at approximately 1150, 1650, and 1750 cm^−1^ correspond to various functional groups including C-O-C stretching of the glycosidic linkage, N-H stretching of amide I, REE-O interactions, and C-O stretching vibrations, respectively [20,21,22,23]. The relative intensities of these peaks show a systematic variation with template loading, with NdPr-AUCH-41 exhibiting the strongest absorbance across these bands. The spectral region between 500 and 700 cm^−1^ reveals characteristic N-H bending vibrations [24]. The most significant spectral differences appear in the fingerprint region (1000–1500 cm^−1^), where imprinted materials show more defined and intense bands, confirming incorporation of both Nd(III) and Pr(III) ions into the polymer matrix.

### 3.3. Thermal Stability Assessment via TGA

**Figure 5:**
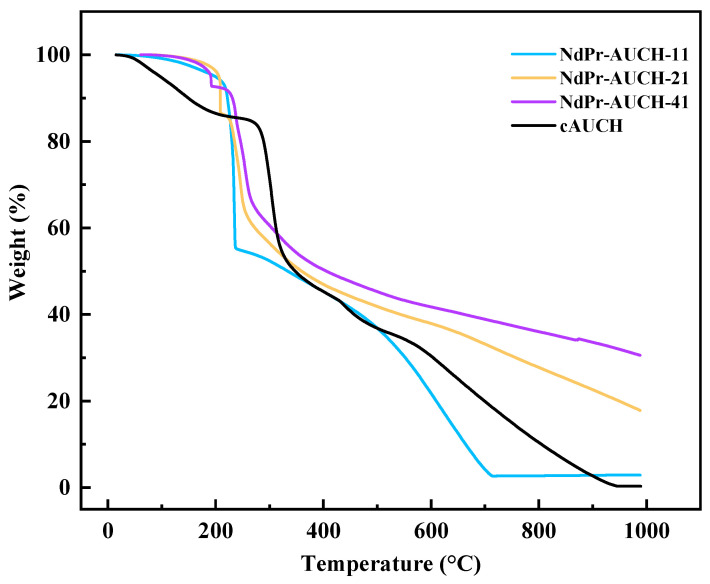
TGA curves of dual-templated AUCH materials and control polymer.

Thermogravimetric analysis was performed to evaluate the thermal stability and decomposition behavior of the dual-templated NdPr-AUCH materials (Figure 5). All samples exhibited a multi-stage decomposition pattern, with initial weight loss (>200 °C) corresponding to the evaporation of bound water molecules from the chitosan network [25]. Decomposition of the chitosan polymer structure occurred in the range 200–400 °C [26]. Beyond 400 °C, the thermal behaviors diverged significantly based on template loadings. NdPr-AUCH-41 demonstrated the highest remaining mass (~30% at 1000 °C), followed by NdPr-AUCH-21 (~18% at 1000 °C), while NdPr-AUCH-11 and the control polymer showed more complete decomposition (~5% and ~0.5% residual mass at 1000 °C, respectively). This systematic increase in remaining mass correlates directly with increasing REE content within the hydrogel, suggesting that higher Nd:Pr loadings contribute to thermal stability, though complete decomposition equilibrium may not have been achieved within the studied temperature range.

### 3.4. Sorption Kinetics and Temperature Effects

**Figure 6:**
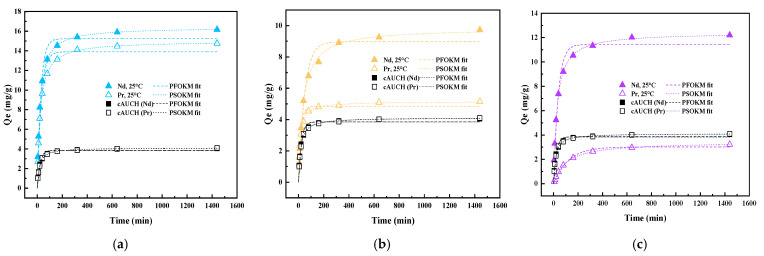
Kinetics of simultaneous Nd(III) and Pr(III) uptake onto AUCH materials at 25 °C (pH = 4, Co = 50 mg/L Nd:Pr). (**a**) NdPr-AUCH-11, (**b**) NdPr-AUCH-21, and (**c**) NdPr-AUCH-41.

**Figure 7:**
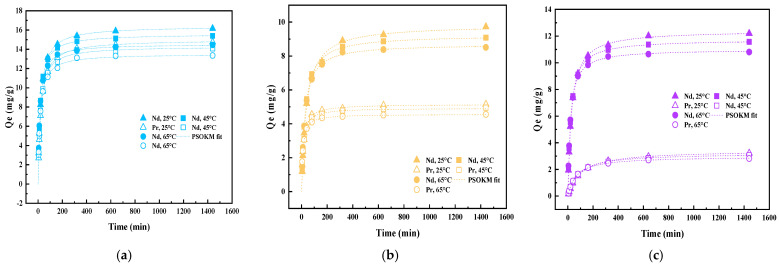
NdPr-AUCH uptake kinetics at various temperatures (pH = 4, Co = 50 mg/L Nd:Pr) (**a**) NdPr-AUCH-11, (**b**) NdPr-AUCH-21, (**c**) NdPr-AUCH-41.

Temperature-dependent kinetic studies (25 °C, 45 °C, and 65 °C at pH = 4) of simultaneous co-uptake of Nd(III) and Pr(III) onto the dual-templated AUCH materials reveal distinct uptake mechanisms that reflect their templating ratios. As shown in Figure 6, all materials exhibited rapid initial uptake, with equilibrium times varying based on template loading. The NdPr-AUCH-11 achieved the highest total REE loading, with maximum capacities of 16.17 mg/g for Nd(III) and 14.75 mg/g for Pr(III) at 25 °C, demonstrating nearly balanced simultaneous uptake. The NdPr-AUCH-21 and NdPr-AUCH-41 displayed increasingly selective Nd(III) binding, with Nd:Pr uptake closely approaching their design ratios (Figure 7). The control material showed minimal, non-selective uptake, while NdPr-AUCH-11 achieved substantially higher capacities with templating-directed 1:1 selectivity, confirming imprinting effectiveness over random sorption. Both pseudo-first-order (PFOKM) and pseudo-second-order (PSOKM) kinetic models were applied to the experimental data. The PSOKM demonstrated superior fits across all systems and temperatures (R^2^ > 0.996), indicating chemisorption-controlled processes. The pseudo-second-order rate constant (k_2_) increased with temperature for all systems, suggesting faster initial uptake rates at elevated temperatures (Table S2).

Activation parameters provided mechanistic insights into the binding processes (Table S3). Activation energies (Ea) ranged from 9.44 to 12.38 kJ/mol, with Pr(III) consistently showing slightly higher values than Nd(III) across all materials. The negative activation entropy (ΔS) values (ranging from −260.79 to −270.41 J/mol·K) indicate increased ordering during complex formation, consistent with inner-sphere coordination mechanisms. The Gibbs free energy of activation (ΔG) increased with temperature for all systems, ranging from 87.41 to 98.24 kJ/mol, characteristic of chemical binding rather than physical sorption.

### 3.5. Effect of Solution pH on Uptake Performance

**Figure 8:**
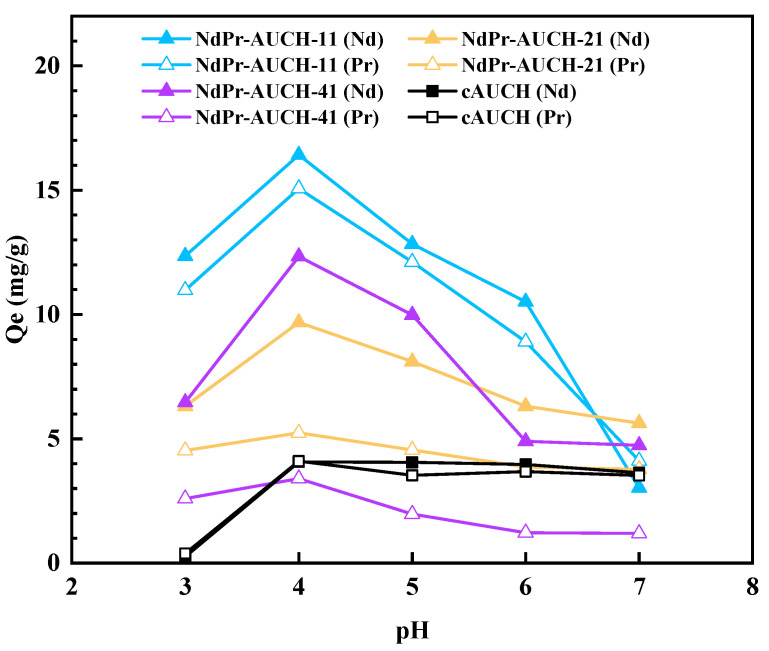
Effect of pH on NdPr-AUCH uptake (25 °C, Co = 50 mg/L Nd:Pr).

The effect of solution pH on the uptake performance of dual-templated AUCH materials was investigated across a pH range of 3–7 (Figure 8). All imprinted polymers demonstrated pH-dependent uptake behavior, with maximum capacities observed at pH 4 and a notable decrease in uptake capacity above pH 5. NdPr-AUCH-11 achieved the highest total REE uptake with simultaneous peak uptake capacities of 16.5 mg/g for Nd(III) and 15.2 mg/g for Pr(III). All materials maintained their designed selectivity ratios most effectively between pH 3–5, with deviation outside that range. The pH 4 optimum balances protonated chitosan amino groups (pKa ~6.5) with minimal REE^3+^ hydrolysis, maximizing electrostatic attraction while maintaining metal ion availability. At optimal pH, the control polymer showed significantly lower uptake capacities for Nd(III) and Pr(III) in all cases, except Pr(III) uptake onto NdPr-AUCH-41.

### 3.6. Sorption Isotherm and Temperature Effects

**Figure 9:**
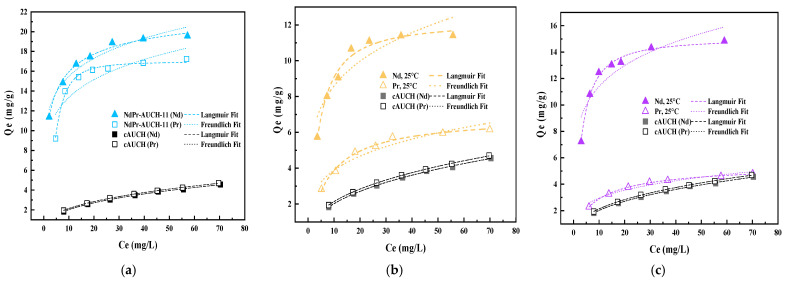
Uptake isotherms of NdPr-AUCH materials and control polymers at 25 °C (pH = 4, Co = 10–75 mg/L Nd:Pr) (**a**) NdPr-AUCH-11, (**b**) NdPr-AUCH-21, (**c**) NdPr-AUCH-41.

**Figure 10:**
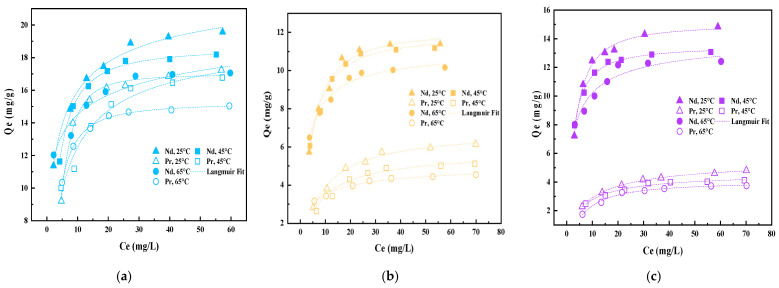
Uptake isotherms of NdPr-AUCH materials at various temperatures (pH = 4, Co = 10–75 mg/L Nd:Pr) (**a**) NdPr-AUCH-11, (**b**) NdPr-AUCH-21, (**c**) NdPr-AUCH-41.

The uptake isotherms for Nd(III) and Pr(III) onto the dual-templated AUCH materials were investigated at 25 °C, 45 °C, and 65 °C. The experimental data were fitted with both Langmuir and Freundlich isotherm models, with the results presented in Table S4. At 25 °C, NdPr-AUCH-11 demonstrated the highest maximum uptake capacities, achieving 19.56 mg/g for Nd(III) and 17.24 mg/g for Pr(III), maintaining a ratio close to its 1:1 templating design (Figure 9). NdPr-AUCH-21 exhibited maximum capacities of 11.40 mg/g and 6.15 mg/g for Nd(III) and Pr(III), respectively, while NdPr-AUCH-41 showed values of 14.83 mg/g and 4.80 mg/g. Figure 10 shows uptake capacities decreased as temperature increased across all materials, consistent with previous work [24]. The Langmuir model provided a better fit across all temperatures and materials, suggesting monolayer uptake on homogenous binding sites.

Thermodynamic parameters were calculated to further understand the uptake mechanisms (Table S5). All systems exhibited negative Gibbs free energy, ranging from −21.58 to −30.77 kJ/mol, confirming the spontaneous nature of uptake. ΔH values were notably lower for Nd(III) than Pr(III) across all dual-templated AUCH materials, suggesting less energy requirements for Nd(III) binding. Positive entropy changes (ΔS) were observed for all systems, suggesting an increased randomness at the uptake interface. This entropy increase likely stems from the displacement of water molecules from both the adsorbent surface and the hydration shells of lanthanide ions during the binding process. The substantial difference in enthalpy values between Nd^3+^ and Pr^3+^ demonstrates the templating process creates distinctly different binding energy environments for these chemically similar ions, providing the thermodynamic basis for selective recognition.

### 3.7. Competitive Sorption

**Figure 11:**
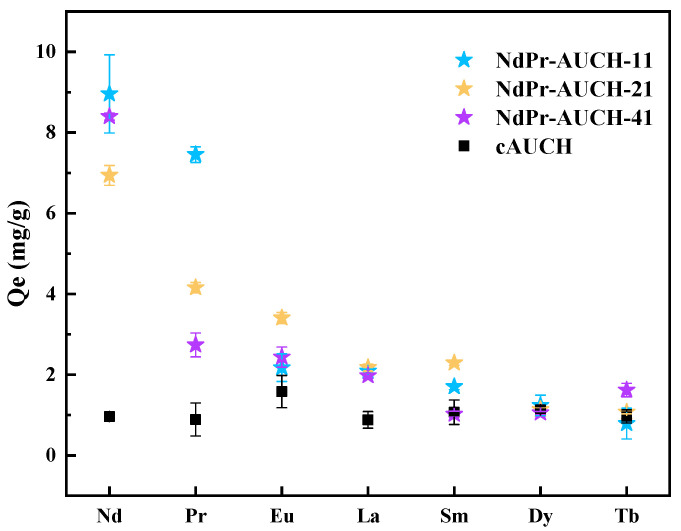
Simultaneous Nd(III) and Pr(III) uptake in the presence of competing REE species via the NdPr-AUCH materials (pH = 4, 25 °C, Co = 50 mg/L for each REE).

The selective recognition capabilities of dual-templated AUCH materials for Nd(III) and Pr(III) were evaluated through competitive uptake experiments in the presence of multiple lanthanide ions. Each polymer was added to a solution containing Nd(III), Pr(III), Eu(III), La(III), Sm(III), Dy(III), and Tb(III) (50 mg/L each, pH = 4) to assess selectivity performance. As shown in Figure 11, NdPr-AUCH-11 demonstrated the highest overall uptake capacity for both target ions, achieving 8.9 ± 0.9 mg/g for Nd(III) and 7.5 ± 0.2 mg/g for Pr(III). NdPr-AUCH-21 showed preferential uptake of Nd(III) over Pr(III), yielding an Nd:Pr ratio of 1.7:1, while NdPr-AUCH-41 exhibited the most pronounced selectivity with 8.4 ± 0.5 mg/g for Nd(III) and only 2.7 ± 0.3 mg/g for Pr(III), resulting in a ratio of 3.0:1. All imprinted polymers displayed significantly lower affinities for the competing lanthanide ions, while cAUCH showed minimal uptake capacities for all lanthanide ions and no selective uptake pattern. The slightly higher Eu^3+^ non-specific binding likely reflects its intermediate ionic radius relative to the Nd^3+^/Pr^3+^ template sizes, creating better geometric compatibility with the binding sites compared to the larger La^3+^/ Sm^3+^ or smaller Dy^3+^/Tb^3+^ ions.

### 3.8. Reusability Studies

**Figure 12:**
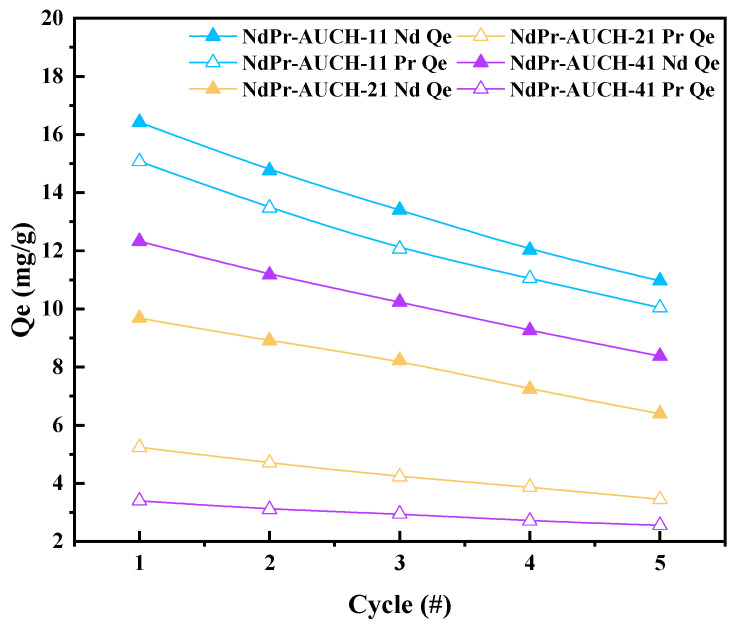
Reusability performance of NdPr-AUCH materials over five adsorption–desorption cycles (pH = 4, 25 °C, Co = 50 mg/L Nd:Pr, 0.05 M EDTA elution).

The practical application of ion-imprinted polymers for REE recovery requires reliable performance over multiple uptake-desorption cycles. To evaluate this aspect, five consecutive uptake-desorption cycles at an initial concentration of 50 mg/L for both Nd(III) and Pr(III) (pH = 4) were conducted for the dual-templated NdPr-AUCH materials. Following each uptake cycle, the materials were eluted with 0.05 M EDTA solution to strip the adsorbed REEs and regenerate the adsorbents for subsequent use. All three NdPr AUCH materials demonstrated decent reusability, maintaining significant uptake capacity after multiple cycles. NdPr-AUCH-11 exhibited the highest retention of initial capacity, preserving 66.8% for Pr(III) and 66.6% for Nd(III) after five cycles. NdPr-AUCH-21 showed similar durability with 65.8% retained capacity for Pr(III) and 66.0% for Nd(III), while NdPr-AUCH-41 demonstrated 75.3% retention for Pr(III) and 67.9% for Nd(III) (Figure 12).

## 4. Discussion

### 4.1. Interface Design: Template Loading Effects on Surface Recognition Properties

The engineered interfaces of dual-template AUCH materials demonstrated selective uptake of Nd(III) and Pr(III) in ratios that closely mirrored their synthesized template loadings, confirming successful surface design for controlled molecular recognition (Table S6). The preservation of these ratios in competitive binding experiments is particularly noteworthy given the chemical similarities between Nd(III) and Pr(III). The SEM analysis revealed only minor morphological differences between materials, suggesting that template ratio modulation does not significantly alter the polymer architecture. Instead, the selectivity likely stems from the precise spatial arrangement of functional groups within the binding cavities, calibrated to accommodate specific Nd:Pr proportions. This interpretation is supported by FTIR analysis. The correlation between template ratio and selectivity persisted across pH 4–5, indicating that the recognition mechanism is robust within acidic conditions.

### 4.2. Interface-Driven Selectivity: Mechanistic Insights from Thermodynamic Analysis

The thermodynamic and kinetic parameters extracted from temperature-dependent studies provide valuable insights into the uptake mechanisms of Nd(III) and Pr(III) onto dual-template AUCH materials. The consistently lower energy requirements for Nd(III) binding compared to Pr(III) across all materials suggests an intrinsic coordination preference, likely stemming from hydration energetics [15]. This energy differential becomes increasingly significant at higher Nd:Pr template ratios, suggesting that the templating process amplifies natural binding preferences through optimized cavity geometries [15,16]. Specifically, Nd^3+^’s smaller ionic radius and higher electronegativity create stronger coordination within the templated sites [15]. While ΔG values fall within the physisorption-chemisorption transition range, the high activation barriers and pseudo-second-order kinetics collectively support coordination-driven recognition mechanisms where differences in hydration energy and electronic configuration influence binding efficiency. The energetic advantage for Nd(III) binding provides the thermodynamic foundation for maintaining template ratio fidelity during simultaneous uptake. These findings suggest potential for further refinement of lanthanide separation strategies based on targeted amplification of small energy differences between chemically similar elements. By precisely engineering binding cavity environments that maximize these energetic differentials, more challenging separations might be achieved.

### 4.3. Alkali/Urea Dissolution System’s Role in Adjacent Lanthanide Discrimination

Traditional chitosan-based materials often struggle to differentiate between chemically similar elements due to heterogeneous binding site distribution and dominant electrostatic interactions [27]. This challenge is overcome in the alkali/urea system, where the disruption of hydrogen bonds during dissolution and subsequent controlled reformation during gelation creates a more homogeneous network structure with uniform binding site distribution [27,28,29]. This restructuring process creates a molecular environment where coordination chemistry can dominate separation processes, enabling the materials to leverage subtle differences in lanthanide electronic structures and complex formation preferences. The minimal surface charge characteristic of alkali/urea dissolved chitosan creates a binding environment where small differences in coordination energetics are less affected by non-specific electrostatic attractions [30,31,32]. The dual-templating approach demonstrates our ability to engineer binding environments with predetermined selectivity patterns, despite inherent performance trade-offs. While this system demonstrated ratio-controlled extraction of adjacent lanthanides, our previous single-template AUCH materials exhibited higher absolute selectivity coefficients and superior regeneration performance over multiple cycles, suggesting that competition between closely related lanthanides for optimized binding sites introduces additional complexity in the coordination environment [18].

## 5. Conclusions

This study demonstrates the successful development of dual-template alkali/urea dissolved chitosan hydrogels capable of simultaneously co-extracting Nd(III) and Pr(III) at ratios that closely mirror their template loading. These novel materials demonstrate remarkable control over selective uptake, with NdPr-AUCH ratios of 1:1, 2:1, and 4:1 successfully translating to corresponding uptake selectivity in controlled environments. Thermodynamic and kinetic analyses reveal lower energy requirements for Nd(III) binding compared to Pr(III), providing mechanistic insights into the template-driven selectivity. This work extends the AUCH platform to the simultaneous separation of adjacent lanthanides, offering new possibilities for rare earth element recovery.

## 6. Patents

A provisional patent has been filed for this technology, and the full application is currently being processed through the University of Missouri System.

## Data Availability

The data presented in this study are available on request from the corresponding author. The data are not publicly available due to privacy.

## Supplementary Materials

The following supporting information can be downloaded at: https://www.mdpi.com/article/10.3390/polym17192567/s1. Table S1: Kinetic and isotherm model equations; Tables S2–S6: Detailed kinetic parameters, thermodynamic data, and selectivity coefficients for all NdPr-AUCH materials.
