# Supplementary material for: Controlled Surface Engineering of Chitosan Hydrogels: Alkali/Urea Dissolution for Ratio-Specific Neodymium and Praseodymium Recovery
Source: Polymers (Basel). 2025 Sep 23;17(19):2567. doi: 10.3390/polym17192567 (PMC12526561; doi:10.3390/polym17192567)
Supplement: Supplementary file 1 [file polymers-17-02567-s001.zip › polymers-3823026-supplementary.pdf]

Supporting Information

Table S1. Summary of equations used in analysis

No.	Description	Equation
1	Adsorption Capacity	$\mathrm{Qe} = \frac{\mathrm{V(Ci\text{-}Ce)}}{W}$
2	Pseudo-First Order Kinetic Model	$\mathrm{qt} = \mathrm{qe}(1\text{-}e^{-k1t})$
3	Pseudo-Second Order Kinetic Model	$\mathrm{qt} = \frac{\mathrm{k2}qe^{2t}}{(1 + k_2 q_e t)}$
4	Langmuir Isotherm	$\mathrm{qe} = \frac{\mathrm{qm{*}KL{*}Ce}}{1 + KL * Ce}$
5	Freundlich Isotherm	$\mathrm{qe} = \mathrm{KF{*}C}e^{1/n}$
6	Thermodynamics	$\Delta\mathrm{G} = -\mathrm{RTln}(K_d)$
7	Gibbs Free Energy	$\Delta\mathrm{G} = \Delta\mathrm{H}\text{-}\mathrm{T}\Delta\mathrm{S}$
8	Activation Energy	$E_a = \mathrm{R}\frac{\ln\left(\frac{k_1}{k_2}\right)}{(1/T_1 - 1/T_2)}$

Table S2. Kinetic model fitting for dual template AUCH materials at various temperatures

Temperature (°C)	Sample	Template	Q,exp (mg/g)	Pseudo-first-order model			Pseudo-second-order model		
				k1 (min-1)	Q,cal (mg/g)	R2	k2 (g/mg*min)	Q,cal (mg/g)	R2
25	NdPr-AUCH-11	Nd	16.17	0.0358	15.24	0.9735	0.0030	16.40	0.9998
45	NdPr-AUCH-11	Nd	15.39	0.0436	14.55	0.9718	0.0039	15.58	0.9997
65	NdPr-AUCH-11	Nd	14.45	0.0502	13.62	0.9641	0.0049	14.54	0.9997
25	NdPr-AUCH-11	Pr	14.75	0.0325	13.90	0.9729	0.0030	15.00	0.9999
45	NdPr-AUCH-11	Pr	14.08	0.0397	13.32	0.9669	0.0039	14.31	0.9984
65	NdPr-AUCH-11	Pr	13.36	0.0449	12.62	0.9564	0.0047	13.52	0.9986
25	NdPr-AUCH-21	Nd	9.72	0.0207	8.97	0.9719	0.0027	9.84	0.9983
45	NdPr-AUCH-21	Nd	9.08	0.0265	8.50	0.9746	0.0038	9.24	0.9990
65	NdPr-AUCH-21	Nd	8.51	0.0297	8.06	0.9718	0.0047	8.71	0.9994
25	NdPr-AUCH-21	Pr	5.14	0.0548	4.85	0.9479	0.0156	5.15	0.9968
45	NdPr-AUCH-21	Pr	4.95	0.0644	4.64	0.9259	0.0194	4.92	0.9948
65	NdPr-AUCH-21	Pr	4.56	0.0744	4.32	0.9312	0.0247	4.56	0.9968
25	NdPr-AUCH-41	Nd	12.19	0.0269	11.44	0.9734	0.0029	12.42	0.9998
45	NdPr-AUCH-41	Nd	11.57	0.0323	10.88	0.9718	0.0038	11.75	1.0000
65	NdPr-AUCH-41	Nd	10.80	0.0380	10.25	0.9727	0.0048	11.00	0.9996
25	NdPr-AUCH-41	Pr	3.21	0.0083	3.02	0.9899	0.0029	3.44	0.9999
45	NdPr-AUCH-41	Pr	3.06	0.0105	2.85	0.9862	0.0040	3.21	0.9999
65	NdPr-AUCH-41	Pr	2.82	0.0123	2.66	0.9875	0.0052	2.97	0.9998

Table S3. Activation Parameters for Sorption Processes

Sample	Activation Parameters	Nd (III)	Pr (III)
NdPr-AUCH-11	Ea (kJ/mol)	10.26	9.44
	ΔH (kJ/mol)	7.62	6.80
	ΔS (J/mol*K)	−267.60	-270.41
	ΔG (kJ/mol) @ 25°C	87.41	87.43
	ΔG (kJ/mol) @ 45°C	92.76	92.83
	ΔG (kJ/mol) @ 65°C	98.11	98.24
NdPr-AUCH-21	Ea (kJ/mol)	11.19	12.16
	ΔH (kJ/mol)	8.55	9.52
	ΔS (J/mol*K)	-265.12	-261.28
	ΔG (kJ/mol) @ 25°C	87.6	87.42
	ΔG (kJ/mol) @ 45°C	92.9	92.65
	ΔG (kJ/mol) @ 65°C	98.2	97.87
NdPr-AUCH-41	Ea (kJ/mol)	10.49	12.38
	ΔH (kJ/mol)	7.86	9.75
	ΔS (J/mol*K)	-267.11	-260.79
	ΔG (kJ/mol) @ 25°C	87.5	87.5
	ΔG (kJ/mol) @ 45°C	92.84	92.72
	ΔG (kJ/mol) @ 65°C	98.18	97.93

Table S4. Isotherm fitting parameters of the dual templated AUCH materials

Temperature	Sample	Template	Langmuir			Freundlich		
			Q,cal (mg/g)	b (L/mg)	R^2	Q,cal (mg/g)	n	R^2
25			19.85	0.327	0.9916	20.47	6.248	0.9544
45	NdPr-AUCH-11	Nd	18.21	0.344	0.9971	19.12	6.791	0.9746
65			17.48	0.208	0.9425	17.68	8.576	0.9332
25			16.97	0.100	0.9919	18.33	5.484	0.7587
45	NdPr-AUCH-11	Pr	17.11	0.227	0.9668	17.71	4.788	0.9183
65			15.02	0.305	0.9977	17.43	7.807	0.8294
25			11.67	0.227	0.9819	12.42	4.667	0.8501
45	NdPr-AUCH-21	Nd	11.39	0.233	0.9841	12.02	4.853	0.8607
65			10.35	0.380	0.9785	10.75	6.368	0.9022
25			6.20	0.129	0.9912	6.52	3.750	0.9245
45	NdPr-AUCH-21	Pr	5.20	0.166	0.9567	5.43	4.089	0.8816
65			4.58	0.292	0.9847	4.68	6.791	0.9593
25			14.68	0.300	0.9928	15.90	5.334	0.8317
45	NdPr-AUCH-41	Nd	13.17	0.444	0.9947	13.94	6.792	0.8374
65			12.79	0.352	0.9347	13.21	6.407	0.8787
25			4.77	0.125	0.9986	4.96	3.747	0.9539
45	NdPr-AUCH-41	Pr	4.17	0.159	0.9778	4.31	4.631	0.9218
65			3.78	0.095	0.9921	4.00	3.784	0.8876

Table S5. Thermodynamic Parameters for Sorption Processes

Temperature	Sample	Template	ΔH (kJ/mol)	ΔS (J/mol·K)	ΔG (kJ/mol)
25					-26.68
45	NdPr-AUCH-11	Nd	1.93	95.95	-28.60
65					-30.52
25					-23.86
45	NdPr-AUCH-11	Pr	23.55	159.01	-27.04
65					-30.22
25					-25.58
45	NdPr-AUCH-21	Nd	10.63	121.43	-28.01
65					-30.43
25					-24.17
45	NdPr-AUCH-21	Pr	16.90	137.77	-26.93
65					-29.69
25					-26.70
45	NdPr-AUCH-41	Nd	3.63	101.71	-28.73
65					-30.77
25					-24.24
45	NdPr-AUCH-41	Pr	9.51	113.19	-26.50
65					-28.76

Table S6. Nd:Pr Adsorption Capacity Ratios per dual templated AUCH material

Test	Material	Temperature	Nd:Pr Qe Ratio
Kinetics	NdPr-AUCH-11	25°C	1.10
	NdPr-AUCH-21		1.89
	NdPr-AUCH-41		3.79
	NdPr-AUCH-11	45°C	1.09
	NdPr-AUCH-21		1.83
	NdPr-AUCH-41		3.78
	NdPr-AUCH-11	65°C	1.08
	NdPr-AUCH-21		1.87
	NdPr-AUCH-41		3.83
pH	NdPr-AUCH-11	25°C	1.09
	NdPr-AUCH-21		1.85
	NdPr-AUCH-41		3.63
Isotherm	NdPr-AUCH-11	25°C	1.14
	NdPr-AUCH-21		1.85
	NdPr-AUCH-41		3.09
	NdPr-AUCH-11	45°C	1.08
	NdPr-AUCH-21		2.18
	NdPr-AUCH-41		3.18
	NdPr-AUCH-11	65°C	1.13
	NdPr-AUCH-21		2.24
	NdPr-AUCH-41		3.32
Competitive Adsorption	NdPr-AUCH-11	25°C	1.20
	NdPr-AUCH-21		1.67
	NdPr-AUCH-41		3.07
Mine Waste	NdPr-AUCH-11	25°C	0.85
	NdPr-AUCH-21		0.91
	NdPr-AUCH-41		2.36
